# A Case of Life-Threatening Airway Obstruction Caused by Acute Diphtheria Infection in the United Kingdom

**DOI:** 10.7759/cureus.19675

**Published:** 2021-11-17

**Authors:** Michael M Chu, Miriam R Bennett, Anna Harrison, Arun Cardozo

**Affiliations:** 1 Otolaryngology - Head and Neck Surgery, Lancashire Teaching Hospitals NHS Foundation Trust, Preston, GBR; 2 Department of Respiratory Medicine, Manchester University NHS Foundation Trust, Manchester, GBR

**Keywords:** diphtheria, corynebacterium ulcerans, airway obstruction, notifiable disease

## Abstract

Diphtheria is a highly contagious and potentially life-threatening infection. Cases in the United Kingdom are rare due to widespread vaccination. However, in recent years, there has been a notable increase in cases in the United Kingdom. We present the case of a 76-year-old British Caucasian female who presented to the Emergency Department with shortness of breath and “chest tightness.” She reported a five-day history of worsening sore throat, odynophagia, and aphonia. On inspection, she had noisy, laboured breathing with the use of her accessory muscles. Flexible laryngoscopy revealed purulent, thick yellow discharge in the nasal cavity, oropharynx, and supraglottis, with oedema of the subglottic mucosa. She became increasingly breathless and was peri-arrest when emergency orotracheal intubation was performed. She was transferred to the Intensive Care Unit for ventilatory support and intravenous antibiotics. Four days after presentation, her microbiology results confirmed toxigenic *Corynebacterium ulcerans*. Public Health England was informed immediately. The patient was isolated and contact tracing was commenced. Thirty staff members were required to self-isolate and take prophylactic antibiotics due to close patient contact. It was particularly noteworthy that our patient was a UK national with no recent history of foreign travel. This case demonstrates the importance of remaining vigilant to atypical causes of airway obstruction secondary to infection. Early suspicion and prompt patient isolation may prevent community and occupational transmission and minimise the impact of contact tracing on hospital staffing. Migration from endemic countries and declining childhood vaccination rates may lead to a further rise in UK cases of diphtheria in the future.

## Introduction

With the advent of widespread vaccination, acute diphtheria infection has become very rare in the United Kingdom. Once known as the “strangling angel” of children, diphtheria was a major cause of childhood mortality in the pre-vaccination era due to the wing-shaped oropharyngeal pseudomembranes causing death by acute airway obstruction [1]. Diphtheria is principally a bacterial respiratory infection in children, but toxins produced by the bacteria can cause potentially lethal cardiac and neurological complications. Toxigenic diphtheria is highly contagious, making it a notifiable disease with potentially serious consequences for public health, occupational health, and hospital workforce staffing. We present the case of an elderly UK national with no history of foreign travel, who presented to a large teaching hospital with airway obstruction secondary to acute diphtheria infection.

This case report was previously presented as an e-poster at the Virtual British Academic Conference of Otolaryngology 2021 on January 10-12, 2021. The case report was also submitted as an assignment by the lead author as a part of a higher degree (MCh Otolaryngology at Edge Hill University, UK).

## Case presentation

A 76-year-old British Caucasian female was brought to the Emergency Department by ambulance with severe shortness of breath. She reported a five-day history of progressively worsening sore throat, odynophagia, and aphonia. She had since become breathless and complained of “chest tightness.” Her medical history included hypertension and ankylosing spondylitis. Her regular medication included ramipril, amlodipine, bendroflumethiazide, atorvastatin, and co-codamol. She had no drug allergies. She was an ex-smoker. There was no history of recent foreign travel.

**Figure 1:**
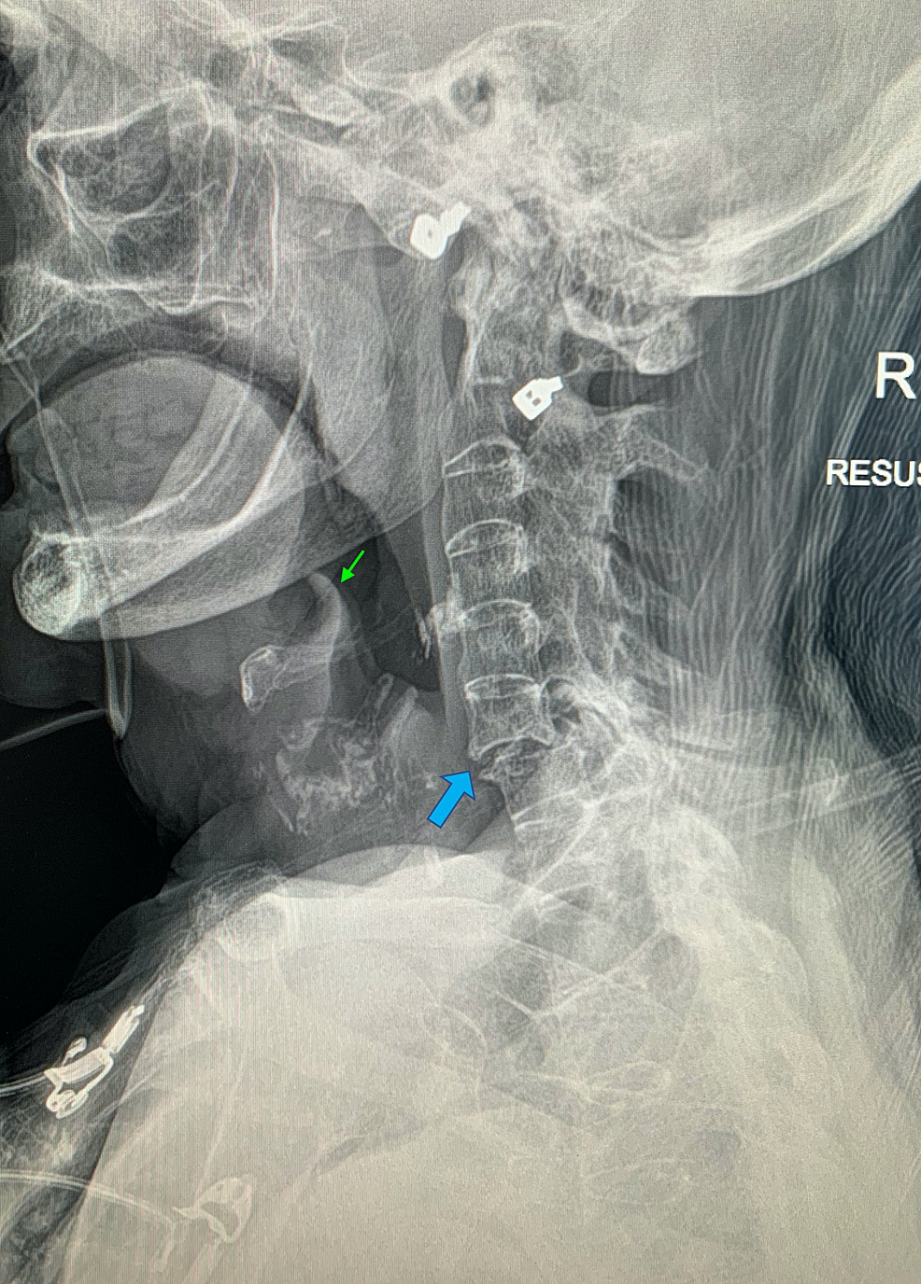
Radiograph of the lateral neck. The image demonstrates normal thickness of the epiglottis (green arrow) with no effacement of the vallecula, degenerative cervical spine secondary to ankylosing spondylitis, fusion of cervical vertebrae C2-C6, and grade 1 anterolisthesis of C6 on C7 (blue arrow).

On initial inspection, there was no stridor, but audible retained secretions were evident on bedside examination. She demonstrated an increased work of breathing with the use of accessory muscles. Consequently, she could only speak in two-to-three word sentences. Her respiratory rate was 28 breaths per minute and her oxygen saturation was 80% on room air. This improved to 96% on 40% fraction of inspired oxygen (FiO_2_). Her heart rate was 134 beats per minute, and her blood pressure 151/88 mmHg. She had low-grade pyrexia of 37.5°C. There was reduced air entry throughout the chest on auscultation. Due to her ankylosing spondylitis, she had very limited neck extension. The thyroid cartilage was palpable, but the cricoid cartilage remained below the suprasternal notch with maximal neck extension. A lateral radiograph of the neck was obtained which showed no evidence of epiglottic swelling (Figure 1). There were degenerative appearances of the cervical spine with a fusion of the C2-C6 vertebrae.

The patient was assessed jointly by the otolaryngology and anaesthetic teams. Flexible nasopharyngolaryngoscopy showed mucopurulent thick yellow secretions filling the entire nasal cavity. The supraglottic mucosa was inflamed and covered with yellow secretions. There was limited abduction of the vocal cords bilaterally. Subglottic mucosal oedema was also noted, signifying potentially impending airway obstruction.

Her blood test showed a raised white cell count of 34 × 10^9^/L and her C-reactive protein was 299 mg/L. Her arterial blood gas showed type one respiratory failure.

She was commenced on intravenous (IV) fluid alongside IV ceftriaxone, IV dexamethasone, and nebulised adrenaline. The patient was immediately transferred to the operating theatre anaesthetic room for definitive airway management. Topical co-phenylcaine was applied intranasally and 10% lidocaine was applied topically to the oropharynx. “Plan A” of awake fibreoptic nasal intubation was attempted, during which the patient became unresponsive and desaturated to 60% despite 60% FiO_2_ via a high-flow nasal cannula. Nasal intubation was abandoned, and the “plan B” of orotracheal intubation was successful using a bougie under awake GlideScope^TM^ (Verathon Inc., Seattle, WA, USA) guidance. A size 6 microlaryngoscopy tube was inserted. “Plan C” was not required, but given her fixed cervical spine and low-lying larynx, front of neck access with emergency cricothyroidotomy was planned instead of tracheostomy. IV propofol, alfentanyl, and metaraminol were commenced. Post-intubation bronchoscopy revealed copious thick membranous secretions in the trachea and both main bronchi.

**Figure 2:**
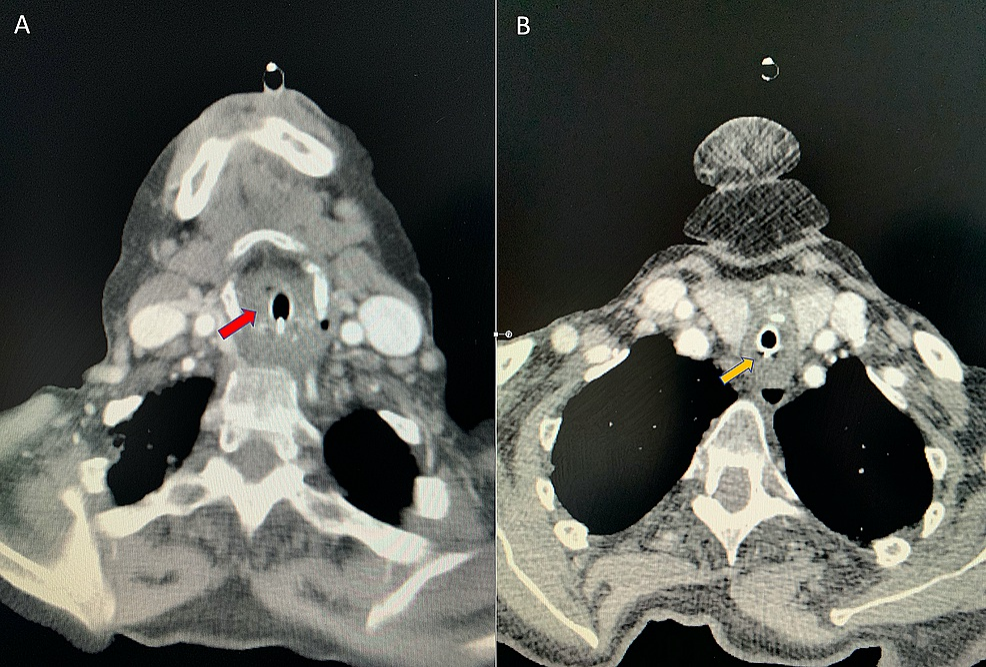
CT of the neck and thorax with contrast. (A) shows laryngeal oedema (red arrow) and (B) shows tracheal oedema (yellow arrow) resulting in severe airway narrowing. The trachea splinted open with an endotracheal tube. CT: computed tomography

Following intubation, the patient underwent a contrast computed tomography (CT) scan of the neck and thorax which showed severe oedema causing significant narrowing of the supraglottic and subglottic airway (Figure 2). A partial left lower lobe collapse with a small left pleural effusion was also noted.

The patient was transferred to the Intensive Care Unit (ICU) for ventilatory and vasopressor support. She was treated with IV cefuroxime and metronidazole alongside IV dexamethasone twice daily. A swab of the nasal secretions was taken and sent for microscopy, culture, and sensitivity. On day four of her admission, the culture showed *Corynebacterium ulcerans*. The sample was then sent to the national reference laboratory to test for toxigenicity. As this was a notifiable infection, the national public health agency, Public Health England (PHE), were informed. They advised that the patient be immediately isolated in a side room. ICU staff were advised to wear appropriate personal protective equipment, with a filtering facepiece 3 mask when performing any aerosol-generating procedures. Her antibiotics were changed to high-dose IV benzylpenicillin and clarithromycin according to sensitivities from the microbiology samples. PHE advised the Occupational Health Department to commence contract tracing for any member of family or staff who had been in close contact and exposed to respiratory droplets within 10 days of the onset of her symptoms. In total, 30 members of the family, medical, and nursing staff were advised to self-isolate at home for one week. Each member of staff had a nasopharyngeal and oropharyngeal swab taken for diphtheria toxin testing. They were offered tetanus, diphtheria, and polio booster vaccine and commenced on prophylactic oral clarithromycin for one week.

Further collateral history was obtained from the patient’s family following the diagnosis of diphtheria. It is unclear whether the patient had been vaccinated against diphtheria during childhood. She lived adjacent to a dairy farm, which had recently flooded. As she was a keen gardener, she would have handled soil on a regular basis. She had several pet dogs that were permitted to roam freely on nearby farmland. She had no known allergies to any animals. She had no history of foreign travel for 20 years.

The patient was given 100,000 units of horse serum-based diphtheria anti-toxin and was monitored closely due to the high risk of anaphylaxis. During her ICU admission, she developed ventilator-acquired pneumonia. Sputum samples grew extended-spectrum beta-lactamase *Escherichia coli* and *Pseudomonas aeruginosa*, requiring a four-week course of IV tazocin, as recommended by the microbiologists. On day 19 of her admission, she had recovered sufficiently to be extubated. She was transferred to the respiratory high-care ward for high-flow oxygen via nasal cannulae and non-invasive ventilation overnight due to type two respiratory failure secondary to pneumonia. The patient did not suffer any cardiac or neurological complications as a direct result of diphtheria infection.

## Discussion

Diphtheria is an infectious disease caused by three main *Corynebacterium* species: *C. diphtheriae*, *C. ulcerans*, and *C. pseudotuberculosis*. These gram-positive rods can produce diphtheria toxin, an exotoxin that causes potentially fatal cardiac and neurological complications if absorbed systemically. *C. diphtheriae*, which only infects humans, is the most common diphtheria strain worldwide [2]. Countries with the highest number of cases include India, Nigeria, and Yemen [3]. *C. diphtheriae *is largely responsible for epidemic person-to-person transmission via respiratory droplets. UK infections due to *C. diphtheriae* are typically imported by patients who have travelled to endemic countries [4].

Although less common globally, *C. ulcerans *has been the predominant strain seen in England in recent years. Of the 10 cases of diphtheria reported to PHE in 2019, all were due to *C. ulcerans* [5]. *C. ulcerans* and *C. pseudotuberculosis* can cause infections in humans and animals. Human cases of diphtheria from these strains are associated with close contact with livestock, domesticated companion animals, and raw dairy products [2]. It is possible that the patient presented in this report contracted *C. ulcerans* from contaminated water supplies from the nearby dairy farm or through contact with her own pet dogs.

**Figure 3:**
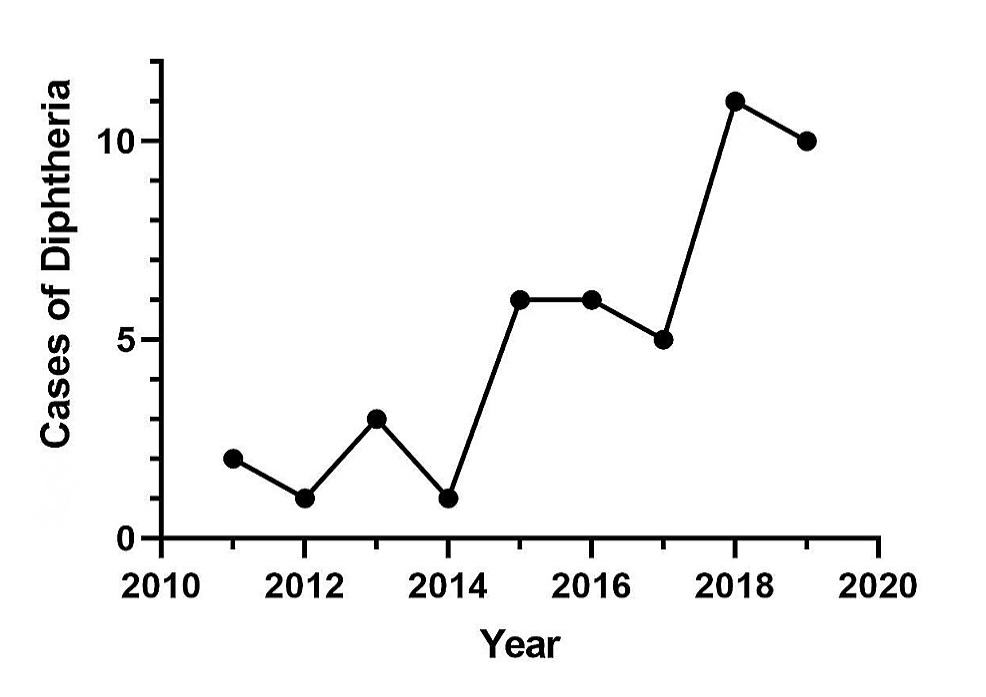
Cases of diphtheria in England (2011-2019) Gathered from Public Health England Data (*C. diphtheriae*, *C. ulcerans*, and *C. pseudotuberculosis* cases included).

Diphtheria appears to be on the rise in England (Figure 3). This mirrors the picture globally, where there has been a year-on-year increase in reported cases for five consecutive years between 2015 and 2019 [6]. Recent humanitarian crises, such as the displacement of Rohingya refugees from Myanmar, demonstrate how disruption of widespread vaccination results in large diphtheria outbreaks [7]. Contrasting reasons have led to a worrying trend of rising cases of vaccine-preventable diseases seen in the developed world. A rise in anti-vaccination movements and populist governments who advocate “medical freedom” have resulted in dwindling vaccination rates in the western world [8]. In unvaccinated populations, the mortality rate of diphtheria is high despite optimal medical management [3].

Diphtheria typically affects children under the age of 15 [3]. It is mainly a respiratory infection, characterised by pseudomembrane formation of the upper aerodigestive tract. This can manifest in severe respiratory distress or even acute airway obstruction causing sudden death [1]. Sore throat and fever are common at presentation. On examination, mucopurulent discharge in the nasal cavity may be present. The patient may have erythema and oedema of the pharyngeal mucosa. There may be extensive cervical lymphadenopathy causing what has previously been described as a “bull neck” appearance [1]. Alternatively, it can present with cutaneous manifestations such as vesicles, pustules, or ulcers, sometimes coated with a pseudomembrane [9].

Definitive airway management (with endotracheal intubation, tracheostomy, or cricothyroidotomy) is required for patients presenting with acute airway obstruction. A multi-disciplinary approach with the involvement of otolaryngology and anaesthetics teams is warranted.

The lethal sequelae of diphtheria are attributed to its toxigenic properties. At a cellular level, diphtheria toxin is taken up by host cells by receptor-mediated endocytosis. It inhibits intracellular protein synthesis, causing cell death which results in macroscopic tissue necrosis [3]. Pseudomembrane formation occurs due to necrosis and subsequent desquamation of the upper airway epithelium [1]. Systemic absorption of diphtheria toxin can cause myocardial damage leading to bradyarrhythmias, tachyarrhythmias, or complete heart block with potentially fatal consequences [3]. Diphtheria toxin can also cause neuronal damage resulting in palatal, pharyngeal, and bulbar paralysis, which causes dysphagia and aspiration. Respiratory muscle paralysis can also occur, which may result in respiratory failure [1].

Given the rapid progression of the disease, treatment should be initiated with empirical antibiotics based on clinical suspicion. Microbiology swabs (nasopharyngeal, oropharyngeal, or cutaneous) should be taken and sent for culture before the first dose of antibiotic. If a pseudomembrane is present, a sample of the membrane should be sent [10]. In England, positive cultures for *C. diphtheriae*, *C. ulcerans*, and *C. pseudotuberculosis* are sent to the national reference laboratory (Public Health England Respiratory and Vaccine Preventable Bacteria Reference Unit, RVPBRU) for confirmation and assessment of toxigenicity [10].

PHE guidelines recommend empirical treatment with macrolides (erythromycin, clarithromycin, or azithromycin) or benzylpenicillin for 14 days [10]. In severe diseases, concurrent macrolide and penicillin therapy can be given while sensitivities are awaited. Post-treatment swabs should be taken to confirm elimination of the organism, and antibiotics should be extended for a further 10 days if not yet eliminated.

Anti-toxin therapy should be administered at the earliest opportunity, but only in confirmed cases or if there is high clinical suspicion of diphtheria. As the anti-toxin is made from horse serum, there is a significant risk of anaphylaxis; hence, the benefits of treatment must outweigh the risks [10]. One systematic review suggested that the administration of anti-toxin reduces mortality by 76% [7]. However, timely administration is crucial as delay can result in high levels of intracellular toxin accumulation. The probability of mortality is 4% if antitoxin is given within 48 hours, which rises to 24% if given at day five or later [7]. The patient described in this case report received her antitoxin dose on day five of her admission. Due to a global shortage of diphtheria anti-toxin, timely procurement can prove challenging [7].

If diphtheria infection is strongly suspected, the patient should be isolated at the earliest opportunity. Due to its notifiable disease status, the governing public health agency must be informed. Contact tracing measures must be undertaken. Family and healthcare workers who have had close contact with the patient in the 10 days preceding symptom onset should be swabbed and advised to self-isolate and commence a one-week course of prophylactic antibiotics. They should be advised to monitor for symptoms of fever, sore throat, and shortness of breath for 10 days. Close contact is defined by family members sharing the same household or healthcare workers subjected to respiratory droplets, for example, during intubation or flexible laryngoscopy [10].

Once clinically stable, the patient should be given a booster dose of diphtheria toxoid-containing vaccine as infection may not result in protective levels of anti-toxin production. The dose should be based on the patient’s age and vaccination history [10]. Full vaccination (with three or more doses) was found to be 87% effective against symptomatic disease and 93% effective in preventing death in one meta-analysis [7]. Immunity from vaccination diminishes over time, with the proportion of individuals with protective antibody titres declining by 0.6% per year since vaccination [7]. Our patient was unsure of her own vaccination status, but it is plausible that she may not have been vaccinated, given she was born in 1943 (mass UK immunisation was introduced in 1942) [5].

## Conclusions

Reported cases of diphtheria in the United Kingdom appear to be on the rise. Diphtheria must be considered in patients presenting with shortness of breath or airway obstruction when copious, thick upper airway secretions are seen. Prompt clinical suspicion and patient isolation may minimise transmission of this highly contagious disease. Typically, diphtheria presents in children and young adults from countries where infections are endemic. However, clinicians should be aware that acute respiratory diphtheria can present in UK nationals with no travel history. In such cases, animal and livestock contact should be interrogated as a part of the clinical history.

## References

[1] Byard RW (2013). Diphtheria - 'The strangling angel' of children. J Forensic Leg Med.

[2] Wagner KS, White JM, Crowcroft NS, De Martin S, Mann G, Efstratiou A (2010). Diphtheria in the United Kingdom, 1986-2008: the increasing role of Corynebacterium ulcerans. Epidemiol Infect.

[3] Sharma NC, Efstratiou A, Mokrousov I, Mutreja A, Das B, Ramamurthy T (2019). Diphtheria. Nat Rev Dis Primers.

[4] Li L, Ross D, Hill K (2020). Two cases of imported respiratory diphtheria in Edinburgh, Scotland, October 2019. Epidemiol Infect.

[5] (2021). Public Health England. Diphtheria in England: 2019. https://assets.publishing.service.gov.uk/government/uploads/system/uploads/attachment_data/file/874090/hpr0620_DPHTHR.pdf.

[6] (2021). World Health Organization. Diphtheria Global annual reported cases and DTP3 coverage 1980-2019. https://www.who.int/immunization/monitoring_surveillance/burden/vpd/surveillance_type/passive/dtp3_global_coverage_2019.jpg.

[7] Truelove SA, Keegan LT, Moss WJ, Chaisson LH, Macher E, Azman AS, Lessler J (2020). Clinical and epidemiological aspects of diphtheria: a systematic review and pooled analysis. Clin Infect Dis.

[8] Hotez P (2019). America and Europe's new normal: the return of vaccine-preventable diseases. Pediatr Res.

[9] Moore LS, Leslie A, Meltzer M, Sandison A, Efstratiou A, Sriskandan S (2015). Corynebacterium ulcerans cutaneous diphtheria. Lancet Infect Dis.

[10] (2021). Public Health England. Public health control and management of diphtheria (in England and Wales). Diphtheria Guidelines Working Group. https://assets.publishing.service.gov.uk/government/uploads/system/uploads/attachment_data/file/774753/Diphtheria_Guidelines_Final.pdf.

